# Deep learning for accurately recognizing common causes of shoulder pain on radiographs

**DOI:** 10.1007/s00256-021-03740-9

**Published:** 2021-02-20

**Authors:** Nils F. Grauhan, Stefan M. Niehues, Robert A. Gaudin, Sarah Keller, Janis L. Vahldiek, Lisa C. Adams, Keno K. Bressem

**Affiliations:** 1 Department of Radiology, Charité – Universitätsmedizin Berlin, Hindenburgdamm 30, 12203 Berlin, Germany; 2 Berlin Institute of Health (BIH), Anna-Louisa-Karsch-Str. 2, 10178 Berlin, Germany; 3 Department of Oral and Maxillofacial Surgery, Charité, Hindenburgdamm 30, 12203 Berlin, Germany; 4 Department of Radiology, Charité, Charitéplatz 1, 10117 Berlin, Germany

**Keywords:** Convolutional neural network, Deep learning, Orthopedics, Shoulder

## Abstract

**Objective:**

Training a convolutional neural network (CNN) to detect the most common causes of shoulder pain on plain radiographs and to assess its potential value in serving as an assistive device to physicians.

**Materials and methods:**

We used a CNN of the ResNet-50 architecture which was trained on 2700 shoulder radiographs from clinical practice of multiple institutions. All radiographs were reviewed and labeled for six findings: proximal humeral fractures, joint dislocation, periarticular calcification, osteoarthritis, osteosynthesis, and joint endoprosthesis. The trained model was then evaluated on a separate test dataset, which was previously annotated by three independent expert radiologists. Both the training and the test datasets included radiographs of highly variable image quality to reflect the clinical situation and to foster robustness of the CNN. Performance of the model was evaluated using receiver operating characteristic (ROC) curves, the thereof derived AUC as well as sensitivity and specificity.

**Results:**

The developed CNN demonstrated a high accuracy with an area under the curve (AUC) of 0.871 for detecting fractures, 0.896 for joint dislocation, 0.945 for osteoarthritis, and 0.800 for periarticular calcifications. It also detected osteosynthesis and endoprosthesis with near perfect accuracy (AUC 0.998 and 1.0, respectively). Sensitivity and specificity were 0.75 and 0.86 for fractures, 0.95 and 0.65 for joint dislocation, 0.90 and 0.86 for osteoarthrosis, and 0.60 and 0.89 for calcification.

**Conclusion:**

CNNs have the potential to serve as an assistive device by providing clinicians a means to prioritize worklists or providing additional safety in situations of increased workload.

## Introduction

Functional limitations of the shoulder due to pain are one of the most common reasons patients seek medical attention and present to the emergency department [1, 2]. While fractures of the proximal humerus and joint dislocation are the most frequent traumatic causes of shoulder pain [3, 4], calcific tendinitis, characterized by calcium depots in the rotator cuffs, is among the most common atraumatic causes [5, 6].

In addition to clinical examination, conventional radiographs of the shoulder are often obtained to identify the underlying causes of shoulder pain, especially in an emergency setting, as they allow swift decisions on further treatment [7]. However, as it may take some time before a radiologist performs the reading, many shoulder radiographs are first viewed by the requesting physician and therapy decisions may be already made at this stage. As requesting physicians are often under time pressure, important findings might initially be overlooked, delaying initiation of adequate therapy for the patients [8]. Therefore, it would be beneficial to have a supportive device, if possible, with very high sensitivity to avoid initial false findings with subsequent treatment delays.

Convolutional neural networks (CNNs) have achieved impressive performances for computer vision tasks on both nonmedical and medical image data, even surpassing human experts [9–11]. The advantages of CNNs are that, once trained, they allow almost instantaneous image interpretation without being susceptible to fatigue or exhaustion. As such, they have great potential in a wide range of applications in the fields of musculoskeletal radiology [12].

The aim of the current study was therefore to train a CNN to detect the most common causes of shoulder pain in plain radiographs, taken in clinical routine, which we defined as fracture, dislocation, calcification, and osteoarthritis (Table 1). Since the presence of osteosynthesis material or an endoprosthesis could affect the accuracy of a CNN, as they are cooccurring, e.g., with fractures, we also aimed for the model to reliably identify them.

**Table 1:** Sensitivity, specificity, and accuracy (with bootstrapped 95% confidence intervals) as well as AUC for detection of the respective findings in the shoulder radiograph after a given threshold was applied to the raw predictions obtained from the model

Type	Sensitivity (95% CI)	Specificity (95% CI)	Accuracy (95% CI)	AUC
Osteoarthritis	0.90 (0.79–0.96)	0.86 (0.80–0.90)	0.87(0.82–0.90)	0.945
Calcification	0.60 (0.43–0.74)	0.89 (0.84–0.92)	0.84(0.79–0.88)	0.80
Dislocation	0.95 (0.76–1.00)	0.65 (0.59–0.71)	0.67 (0.61–0.73)	0.896
Fracture	0.75 (0.61–0.85)	0.86 (0.81–0.91)	0.84(0.79–0.88)	0.871

## Methods

### Data preparation

A total of 3682 plain radiographs were extracted from multiple institutions serving PACS (Picture Achieving and Communication System), then transferred to a separate server and converted from DICOM format (Digital Imaging and Communications in Medicine) to Tagged Image File Format (TIFF). Images not containing the shoulder were manually excluded (*n* = 38); otherwise, no further preselection of the images was done. The resulting dataset included 3644 radiographs acquired from different views such as anterior-posterior view, Y-view, or outlet view but also non-standardized images taken during surgical procedures or in the emergency room.

### Distribution of findings

**Fig. 1:**
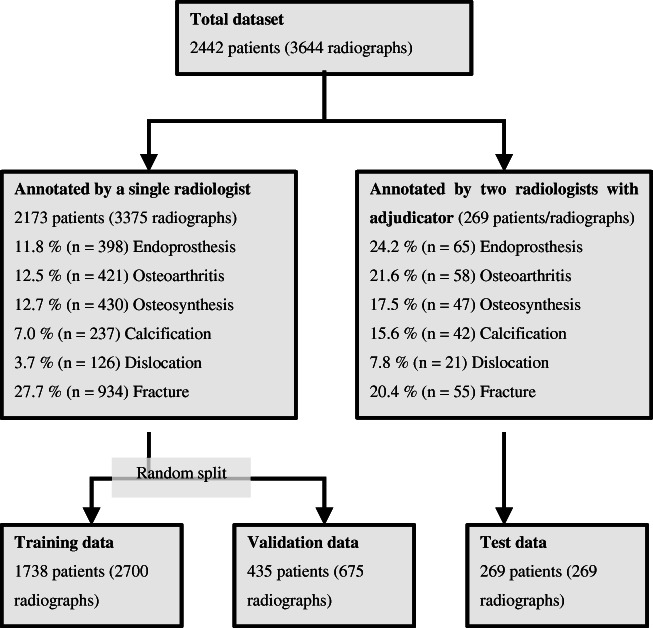
Flowchart of the data selection process and the composition of the final datasets as well as an overview of the frequencies of different findings

A total of 3644 radiographs from 2442 patients were included in this analysis. The distribution of findings in the whole dataset was strongly skewed. The most common finding was fracture in 27.1% of cases (*n* = 989), followed by osteoarthritis (13.1% of cases, *n* = 479), osteosynthesis (13.1% of cases, *n* = 477), endoprosthesis (12.7% of cases, *n* = 463), calcification (7.7% of cases, *n* = 279), and dislocation (4.0% of cases, *n* = 147). 26.8% of all radiographs (*n* = 978) showed no pathological finding. An overview of label distribution and the data selection process is provided in Fig. 1. The distribution of findings is summarized in Table 2.

**Table 2:** Summary of the distribution of findings in training, validation, and test dataset as well as in the whole dataset. The numbers between the radiographs and the captions are different because multiple captions were allowed to a single image

	Training dataset	Validation dataset	Test dataset	Whole data
No finding	799 (29.6%)	172 (25.5%)	7 (2.7%)	978 (26.8%)
Fracture	771 (28.6%)	163 (24.1%)	55 (20.4%)	989 (27.1%)
Dislocation	109 (4.4%)	17 (2.5%)	21 (7.8%)	147 (4%)
Calcification	152 (5.6%)	85 (12.6%)	42 (15.6%)	279 (7.7%)
Endoprosthesis	302 (11.1%)	96 (14.2%)	65 (24.2%)	463 (12.7%)
Osteosynthesis	354 (13.1%)	76 (11.3%)	47 (17.5%)	477 (13.1%)
Osteoarthritis	326 (12.1%)	95 (14.1%)	58 (21.6%)	479 (13.1%)

### Data labeling and splitting

From this extracted collection of images, three datasets were generated for training, validation, and testing of the model.

First, all images were labeled by a radiologist. This allowed us to group the images by label and randomly draw from different groups of images when curating the test dataset. We chose this method to easily correct for rare labels in the test dataset, as very low prevalence may negatively affect the calculation of accuracy. The test dataset was curated and consisted of *n* = 269 radiographs, and we took care to include only one radiograph per patient. All 269 images were again labeled by a single radiologist who was blinded to the previous labels. In cases where the first and second radiologists disagreed, a third radiologist served as a reviewer.

Then, the remaining 3375 radiographs were used to create the training and validation dataset. We used a random split of 80–20, resulting in a training dataset with *n* = 2700 radiographs and a validation dataset with *n* = 675 images. Figure 1 shows a flowchart of the data extraction process.

All annotations were performed using the open source program labelImg (https://pypi.org/project/labelImg/). Annotation included the following labels: fracture, dislocation, calcification, osteoarthritis, osteosynthesis, and total endoprosthesis. Multiple tags to one image were allowed. Annotations were then exported into the XML format (Extensible Markup Language) and converted to one-hot encoded annotations in tabular format using the “R” programming language and the “tidyverse” library [13].

### Model training

We employed a convolutional neural network of the Resnet architecture with 50 layers, pretrained on the ImageNet dataset [14]. For implementation, the Python programming language (https://www.python.org, version 3.8) with the PyTorch (https://pytorch.org) and FastAI (https://fast.ai) libraries was used on a workstation running on Ubuntu 18.04 with two Nvidia GeForce RTX 2080ti graphic cards (11 GB of RAM each).

Prior to any training, the images were scaled down to a width of 1024 pixel (px), maintaining aspect ratio. Pixel values of the images were normalized, and the data were augmented through random rotations, mirroring, and distortion of the images. We used a progressive resizing approach, starting at an image size of 64 × 64 px and a batch size of 256 images for a total of ten epochs with discriminative learning rates (meaning the learning rate was lower for the first layers of the network than for the last layers). For the first five epochs, only the classification head of the model was trained with the remaining weights remaining unchanged, while for the last 5 epochs, all weights of the model were updated. After each training session, image resolution was successively increased to 128 × 128, 256 × 256, 512 × 512, and finally 768 × 768 px. Used batch sizes were 256 for 64px, 128 for 128 px, 64 for 256px, 32 for 512 px, and 16 for 768 px.

For the last ten epochs of training at 768 px image size, checkpoints were saved after each epoch. Finally, each checkpoint was evaluated on the test dataset, and the predictions of the ten checkpoints were pooled to calculate accuracy.

For the purpose of illustrating predictions from the model, exemplary Gradient-based Class Activation Mappings (Grad-CAM, [15]) were calculated. Example code for model training is available at https://gist.github.com/kbressem/7e6fb4ec270cbacde07a379562a18a4b.

### Statistical analysis

Predictions on the test dataset were exported as comma-separated values (CSV) and then further analyzed using the “R” statistical language and the “tidyverse,” “irr,” and “ROCR” libraries [16–19]. Model performance was evaluated using receiver operating characteristic (ROC) curves and calculating the area under the curve (AUC). Interrater agreement was evaluated using Cohen’s kappa. 95% confidence intervals (95% CI) were calculated through bootstrapping with 1000 iterations.

## Results

### Model accuracy

**Fig. 2:**
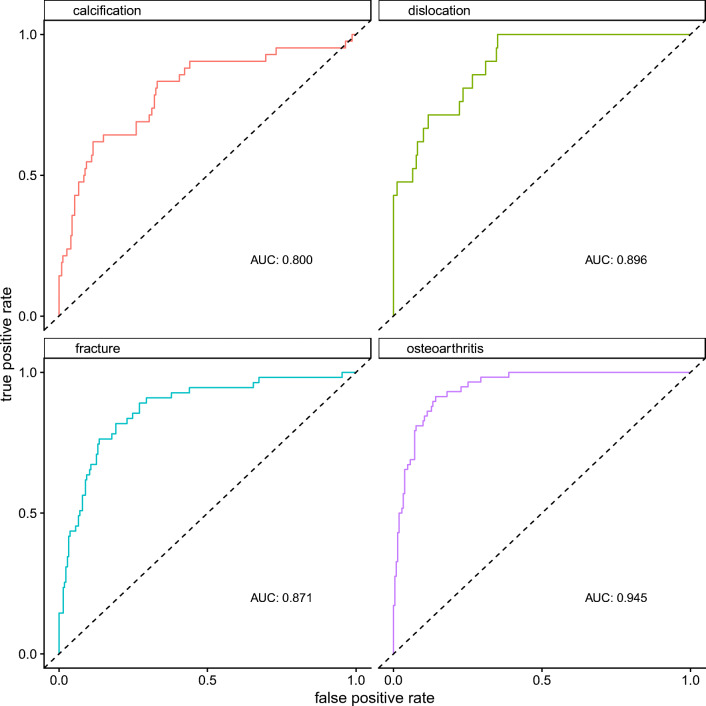
Receiver operating characteristics curves with the respective areas under the curve (AUCs) for the detection of the six different findings. For osteoarthritis, the model achieved the best performance with an AUC of 0945, while detection of calcifications posed a stronger challenge for the model as suggested by an AUC of 0.8

The model showed the highest accuracy for the detection of osteoarthritis with an area under the receiver operating characteristics curve (AUC) of 0.945 (95% CI 0.917–0.972). For dislocation, fracture, and calcification, accuracies were lower with AUCs of 0.896 (95% CI 0.836–0.956), 0.871 (95% CI 0.817–0.926), and 0.80 (95% CI 0.717–0.883), respectively. Receiver operating characteristics curves and AUCs are presented in Fig. 2. The presence of a shoulder endoprosthesis and osteosynthesis material were detected with near perfect accuracy (AUC of 1.00 (95% CI 0.99–1.00) and 0.998 (95% CI 0.993–1.00)).

**Fig. 3:**
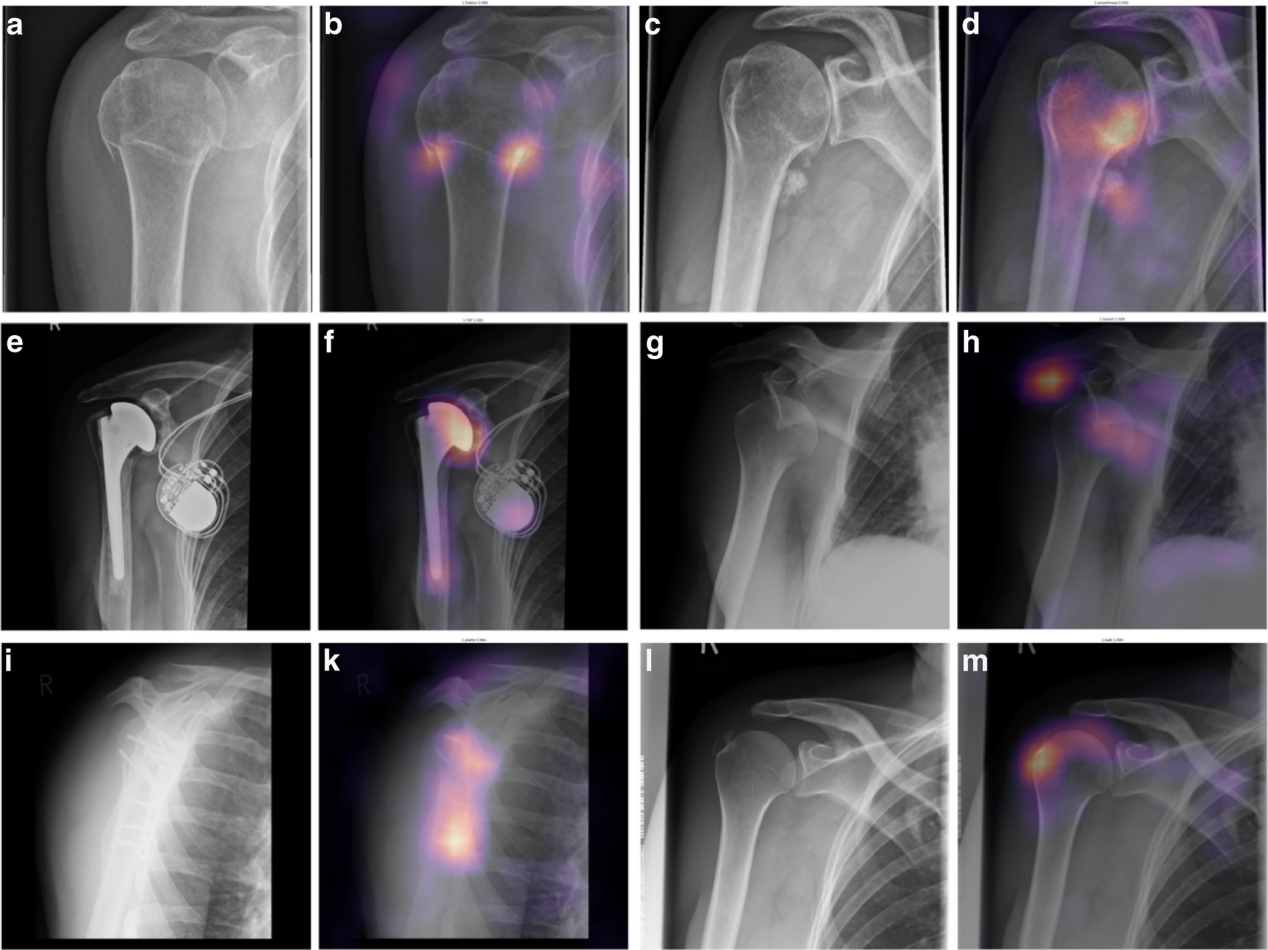
Examples of correct predictions highlighted by Gradient-weighted Class Activation Mappings (Grad-CAMs) for radiographs with only one finding. Images A, C, E, G, I, and L are the original images showing a fracture (A), osteoarthritis (C), an endoprosthesis (E), dislocation (G), osteosynthesis material (I), and calcification (L). Images B, D, F, H, K, and M are the corresponding Grad-CAMs. Grad-CAMs highlight image areas that were important to the model’s decision. They can be used to verify that a pathology was correctly identified and that the decision was not based on image noise (e.g., due to overfitting) or a different finding (e.g., a model that should detect a fracture but instead detects only osteosynthesis)

**Fig. 4:**
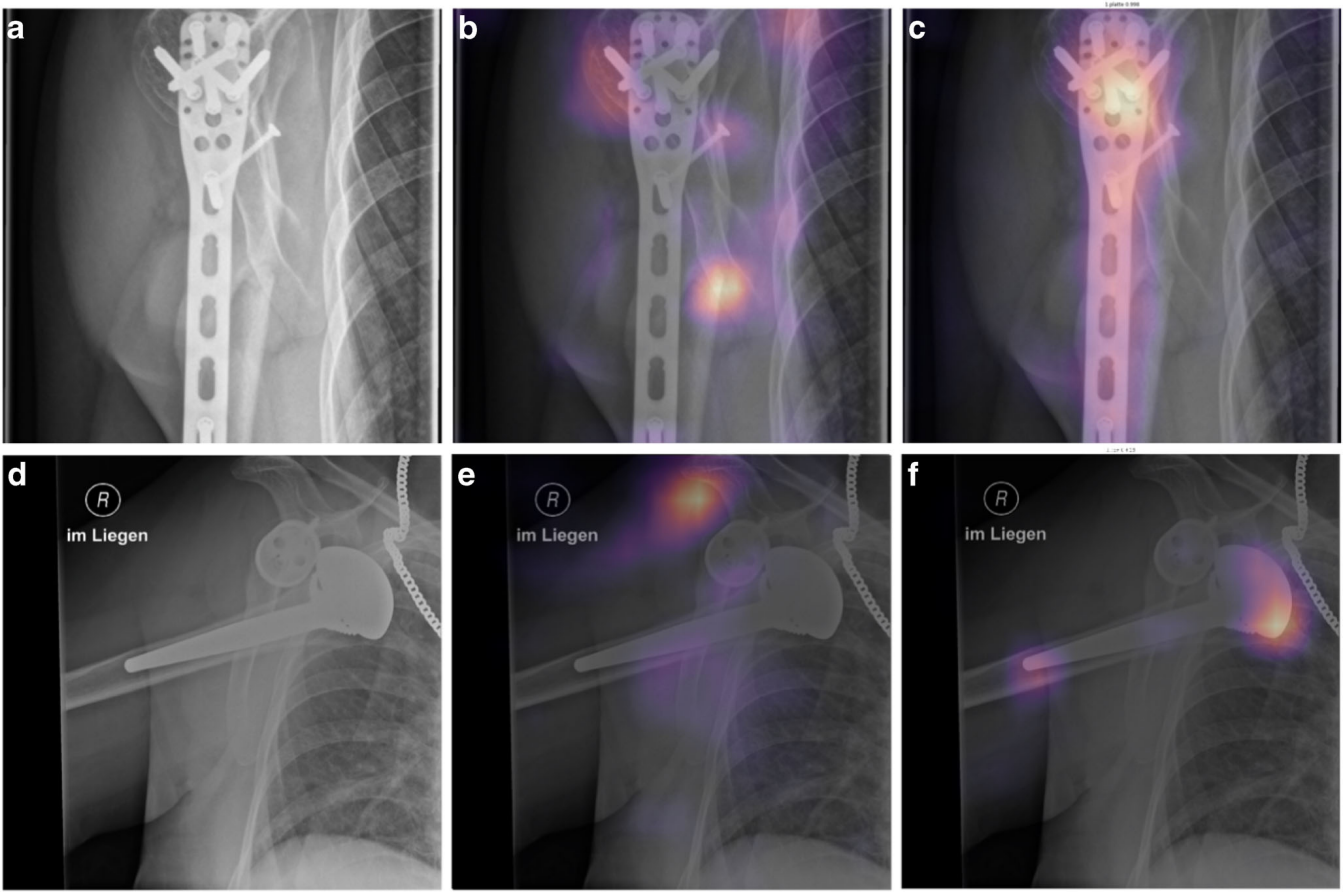
Examples of correct predictions highlighted by Gradient-weighted Class Activation Mappings (Grad-CAMs) for multilabel classifications. A and D are the original images, showing a fracture at the humeral head and the humeral shaft with osteosynthesis material (A) and a dislocated shoulder endoprosthesis (D). In B, the model showed a strong activation in image regions of the fracture and in C a strong activation for image regions depicting osteosynthesis material, which lead to the models final classification result. E shows activation of the model in the area where the humeral head should be, leading to the recognition of the dislocation, and F shows the activation for detection of the endoprosthesis

Sensitivity, specificity, and accuracy were calculated choosing a threshold for predictions so that the sum of sensitivity and specificity was highest. Sensitivity/specificity/accuracy were 0.90/0.86/0.87 for osteoarthritis, 0.75/0.86/0.84 for fractures, 0.95/0.65/0.67 for joint dislocation, and 0.60/0.89/0.84 for periarticular calcification. Sensitivity, specificity, accuracy, and AUC results are also compiled in Table 1. Examples of Grad-CAMs are shown in Figs. 3 and 4.

### Interrater reliability

Interrater reliability between the two radiologists on the test dataset was moderate and 78 of 269 radiographs needed adjudication by a third reader. For fracture, a kappa value of 0.61 (95% CI 0.50–0.73) was achieved, which was the second highest agreement. For calcification, dislocation, and osteoarthritis, agreement was even lower with kappa values of *k* = 0.59 (95% CI 0.45–0.74) for calcification, *k* = 0.53 (95% CI 0.32–0.74) for dislocation, and *k* = 0.47 (95% CI 0.33–0.60) for osteoarthritis.

## Discussion

The aim of the present study was to train a CNN to detect the most common causes of acute or chronic shoulder pain in plain radiographs. The proposed CNN shows good overall accuracy, suggesting that such networks have the potential to provide additional help and security for the physician on duty.

**Fig. 5:**
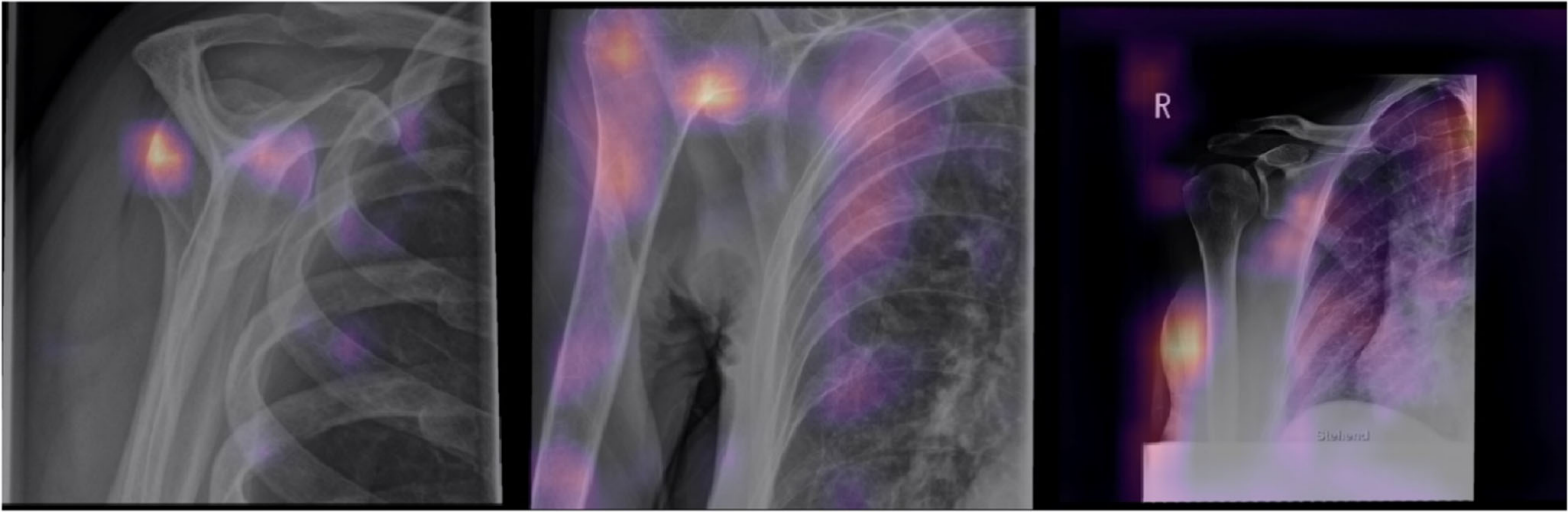
Examples of incorrect predictions. The left images are falsely labeled as calcification. The middle image shows an incorrectly labeled osteoarthritis and the right image shows an incorrectly predicted fracture. All of the images are atypical images, which may have been one of the causes of the incorrect predictions. For example, in the right image, there is some activation at the sharp edges of the image, which may have led the algorithm to predict a fracture. In the middle image, larger portions of the joint plane are not visible, which was a possible cause of the incorrectly predicted osteoarthritis

Despite a rapid increase in published studies on medical image classification, so far only a few investigators have focused on shoulder radiographs and causes of shoulder pain. To the best of our knowledge, there is only one comparable study by Chung et al., in which the authors proposed a CNN explicitly for the detection of proximal humeral fractures [20]. In their approach, Chung et al. focused on the classification of different fracture types, achieving high accuracies, as measured by the AUC. However, they only included shoulder radiographs acquired in strict anterior-posterior patient position and thus did not cover the full range of variability encountered in clinical practice. We are convinced that the inclusion of images acquired under various non-standardized conditions results in a more robust method that is less susceptible to errors in everyday use (Fig. 5).

Recently, two separate studies have demonstrated the efficiency of CNNs in detecting and even differentiating shoulder implants by the manufacturer [21, 22]. However, these studies relied on frontal view radiographs, whereas our data included a broad variety of views to allow detection of surgical implants independent of image settings used for acquisition. Therefore, our model may prove to be more robust on clinical data, which include radiographs of suboptimal image quality, as pain may preclude optimal patient positioning.

Dislocated shoulder joints were reliably detected by the model. From our point of view, this is essential for a real-life approach since shoulder joint dislocation is the most common type of dislocation in humans [4].

Although still detected with high accuracy, it was periarticular calcifications that posed the greatest challenge for our model. Intuitively, this is not surprising, especially since small calcium deposits can be partially obscured by bone spurs in osteoarthritis or are difficult to distinguish from small fragments. To our knowledge, this is the first study that also focuses on periarticular calcifications.

There are several studies in the literature that have trained neural networks to recognize hip and knee joint osteoarthritis [23–25]. None of these studies included the shoulder joint. Although osteoarthritis of the shoulder joint is less common in the general population, it is a frequent and therefore relevant trauma sequel in the post-injury patient population [4, 26]. Therefore, detecting this finding with high accuracy, as our model does, is important for any future clinical implementation.

We used a multiclass classification model that could predict all classes for each image instead of using multiple binary classifiers. This is similar to the approach used by von Schacky et al. [27], who developed a multiclass classification model for osteoarthritis detection. Other studies used network architectures originally developed for object detection. Here, classification is combined with highlighting of relevant areas using bounding boxes. This approach was successfully applied by Krogue et al. [28], who used a DenseNet to identify and subclassify hip fractures.

Overall, there are many practical reasons to continuously improve neural networks, which have the potential to become valuable supportive devices for medical staff in the future. The most important help these networks can provide could be error reduction, especially in situations of increased workload, and worklist optimization, prioritizing critical findings or serving as a second reader. A different, and basically simplified, approach in order to achieve this goal was used in the work by Rajpurkar et al. [29]. In their study, the authors trained a neural network for the simple binary classification task of differentiating between normal and abnormal images from the publicly available MURA dataset (a collection of conventional images of the upper extremity). Interestingly, they found that their model performed worse than its human counterparts on shoulder images, while it achieved comparable results on images of the wrist, hand, and fingers. We conclude that plain radiographs of the shoulder may pose a considerable challenge to CNNs, especially when they are expected to detect more subtle findings such as periarticular calcification. Overall, however, our results are promising and strongly encourage further trials ultimately leading to clinical implementation.

## Limitations

Our study has several limitations: No additional clinical information about the population included in the study was retrieved, posing a risk of bias through possible imbalance in patient characteristics (e.g., age, sex, medical preconditions). Additionally, only a limited number of labels were assessed and did not include any form of grading pathological findings such as the severity of osteoarthritis or the specific type of a fracture. In its current state, this would likely hinder clinical implementation. Furthermore, the ground truth was solely based on reading radiographs (instead of using more robust resources such as follow-up cross-sectional imaging) and labeling training and validation data were performed by a single radiologist. Both measures carry the risk of bias and mislabeling. Also, computational capacity put a limit on the applied image resolution, potentially impairing model performance when faced with subtle findings such as small calcifications or bone fragments.

## Conclusion

The rapid evolution of CNNs in medical imaging is paving the way for the development of assistive devices that may eventually help medical personnel by providing a second reading instance. We present a robust CNN with the ability to detect common causes of shoulder pain on plain radiographs, even when faced with impaired image quality as often seen in clinical practice, especially in emergency settings.
